# Photo-induced transformation process at gold clusters-semiconductor interface: Implications for the complexity of gold clusters-based photocatalysis

**DOI:** 10.1038/srep22742

**Published:** 2016-03-07

**Authors:** Siqi Liu, Yi-Jun Xu

**Affiliations:** 1 State Key Laboratory of Photocatalysis on Energy and Environment, College of Chemistry, Fuzhou University, Fuzhou, 350002, P. R. China; 2 College of Chemistry, New Campus, Fuzhou University, Fuzhou 350108, P. R. China

## Abstract

The recent thrust in utilizing atomically precise organic ligands protected gold clusters (Au clusters) as photosensitizer coupled with semiconductors for nano-catalysts has led to the claims of improved efficiency in photocatalysis. Nonetheless, the influence of photo-stability of organic ligands protected-Au clusters at the Au/semiconductor interface on the photocatalytic properties remains rather elusive. Taking Au clusters–TiO_2_ composites as a prototype, we for the first time demonstrate the photo-induced transformation of small molecular-like Au clusters to larger metallic Au nanoparticles under different illumination conditions, which leads to the diverse photocatalytic reaction mechanism. This transformation process undergoes a diffusion/aggregation mechanism accompanied with the onslaught of Au clusters by active oxygen species and holes resulting from photo-excited TiO_2_ and Au clusters. However, such Au clusters aggregation can be efficiently inhibited by tuning reaction conditions. This work would trigger rational structural design and fine condition control of organic ligands protected-metal clusters-semiconductor composites for diverse photocatalytic applications with long-term photo-stability.

Aiming at global energy crisis and environmental contamination caused by the overuse of fossil fuels, renewable photocatalytic solar energy conversion holds great promise to be an attractive alternative technology to burning nonrenewable fossil fuels^1^,^2^,^3^,^4^. A significant challenge in the research of photocatalytic solar energy conversion is the rational design of photocatalysts that can efficiently harvest solar energy, convert into charge carriers, and allow controlled transfer of those charges for participating in redox reactions^5^. Loading of metal nanoparticle cocatalysts that behave as reaction sites on the surface of the photocatalyst is an effective strategy to promote the photocatalytic process efficiency^6^,^7^. In addition, metal nanoparticles, particularly gold nanoparticles, are well-known for their characteristic plasmon absorptions in the visible light region, which attracts significant interest in metal plasmon based photocatalysis^8^,^9^. Furthermore, the low-lying Fermi level endows metal nanoparticles with the capability of acting as an electron sink for decreasing the recombination rate of photo-generated charge carriers^10^,^11^. Smaller metal nanoparticles with precise atomic control, often called metal clusters, have more discrete molecule-like states and show rich optical, electrochemical, and other physicochemical properties^12^,^13^,^14^,^15^,^16^. When metal clusters can be made with very small sizes comparable to the Fermi wavelength or the deBroglie wavelength of the conduction electrons (around 2 nm), they exhibit the prominent characteristics that are different from those of both bulk and large nanoparticles owing to the quantum confinement effects on electrons^17^,^18^,^19^. Therefore, significantly growing interest has been devoted to studying the metal clusters based (typically the coupling composites of metal clusters with semiconductors) photocatalysis^20^,^21^,^22^,^23^,^24^.

Among them, organic ligands protected gold clusters (Au clusters) have attracted extensive research interests and found broad applications in biomedicine, sensing, optoelectronics, and catalysis, due to their unique physicochemical properties arising from a small core size and heavy ligation by organic ligands^25^,^26^. Most recently, Au clusters have emerged as a type of novel photosensitizer, which is able to extend the light response and photo-activity of wide band-gap semiconductors^16^,^27^,^28^. These Au clusters with few dozen metal atoms in the core with bound organic ligands in the shell possess unique atom-packing mode, strong electron energy quantization induced by the ultra-small cluster size, sizable band-gap, and controllable catalytic properties^29^. Their photophysical properties resemble those of molecular organometallic complexes, thus making Au clusters offer the flexibility in photocatalytic systems similar to semiconductor quantum dots^30^. In addition, Au clusters have also been shown to directly catalyze charge transfer processes^31^. The combination of photo and catalytic activity makes Au clusters an ideal choice for the multifunctional use in photocatalytic systems as they can serve as both photosensitizer and catalytic center^28^.

It is well known that the photophysical properties of Au clusters can be tuned through their composition, size, and ligands^29^. The organic ligands can help to stabilize Au clusters against aggregation and play an important role in surface functional modification of Au clusters^32^,^33^,^34^,^35^,^36^. Since the photo-generated holes and/or their derivative oxidative species always exist in the photocatalytic systems, the photo-induced oxidative chemical and structural transformations of organic ligands protected-Au clusters under practical operative conditions would have a great impact on the size, optical, photoelectrochemical (PEC) properties and the photocatalytic performance of Au clusters-semiconductor composites. Seminal works about Au clusters-semiconductor-based photocatalysis with short-term performance enhancement have been reported^5^,^7^,^23^,^27^. However, in the long view, the question that one encounters is whether the organic ligands protected-Au clusters can withstand the onslaught of highly reactive oxygen species (e.g., hydroxyl and superoxide radicals) which exist in the photocatalytic system. Although instability of gold nanoparticles at the metal/semiconductor interface under the UV light irradiation has been observed in previous study^37^, a systematic and exhaustive investigation on the photo-stability of organic ligands protected-Au clusters at the metal/semiconductor interface and its influence on photocatalytic performances and mechanism of Au clusters-semiconductor composites should be unambiguously established.

With these motivations, herein, we have fabricated glutathione-capped Au clusters modified TiO_2_ (Au GSH clusters-TiO_2_) composites as a representative example for investigating the photo-stability of organic ligands protected-Au clusters at the metal/semiconductor interface. Its influence on the photocatalytic performances and reaction mechanism is also studied. It is found that an oxidative transformation of ultra-small molecular-like Au GSH clusters to larger metallic plasmonic nanoparticles with a diffusion/aggregation process can be triggered under both the simulated solar light and visible light irradiation *via* different oxidation mechanisms. The existence of both plasmonic Au nanoparticles (Au NPs) and molecular-like Au GSH clusters during the photo-induced transformation process leads to the different photocatalytic mechanism from that for Au GSH clusters-TiO_2_ composites, which highlights the complexity of Au GSH clusters-semiconductor composite based photocatalysis. Similar photo-induced aggregation have also been observed in the most studied highly stable Au_25_(SG)_18_ clusters, which further confirms the instability of organic ligands protected-Au clusters at the metal/semiconductor interface during the long-term photo-irradiation under practical operative conditions. In addition, a series of controlled experiments have demonstrated that this photo-induced aggregation of Au GSH clusters can be efficiently inhibited by finely controlling the reaction conditions. It is hoped that this work could raise the significant concern on the judicious use of metal clusters in diverse photocatalytic solar energy conversions and promote the rational structural design and reaction condition control toward the purpose of achieving metal clusters-semiconductor composite photocatalysts with long-term photo-stability for target redox applications.

## Results

### Characterization of Au GSH Clusters

**Figure 1:**
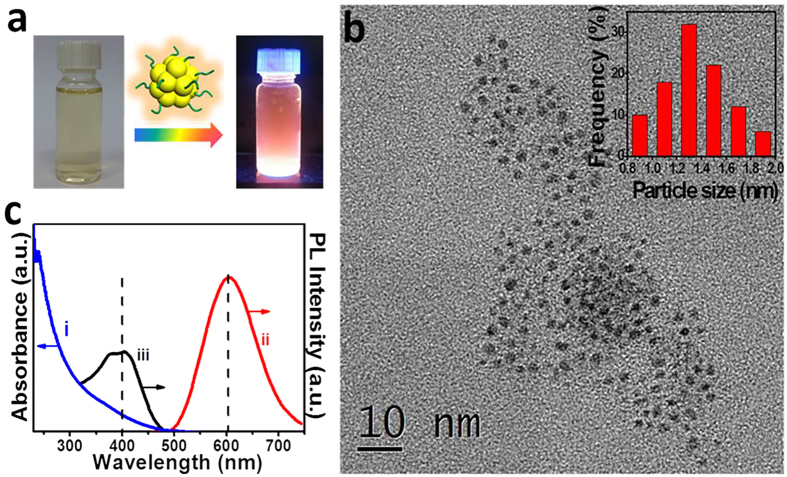
Morphology and optical properties of Au GSH clusters. Molecular model and digital photograph of Au GSH clusters aqueous solution under the daylight and blacklight illumination, respectively (**a**) TEM image and size distribution histogram (inside) of Au GSH clusters (**b**) and (i) UV-vis absorption, (ii) emission spectrum and (iii) excitation spectrum of Au GSH clusters in aqueous suspension (**c**). The excitation wavelength was 400 nm for recording the emission spectrum. Excitation spectra were recorded by monitoring emission at 605 nm. Excitation and emission spectra were normalized to the 400 nm absorption.

Emissive yellow solution under the blacklight illumination indicates the successful formation of glutathione-protected Au clusters (Au GSH clusters) (Fig. 1a)^26^,^38^. Transmission electron microscopy (TEM) has been utilized to directly examine the formation of Au GSH clusters. As shown in Fig. 1b, mean diameter of the as-synthesized Au GSH clusters is determined to be around 1.4 nm, which indicates the formation of rather small metal clusters with a few-dozen Au atoms^26^,^38^. Due to the unique atom packing structure and quantum confinement effects, it is quite difficult to distinctly observe the crystal lattice by the high-resolution TEM (HRTEM) analysis (Supplementary Fig. S1). According to the previous reports^26^,^27^,^38^, these Au GSH clusters are demonstrated to possess Au(0)@Au(I)-thiolate core-shell morphology with relatively high concentration of thiolate ligands (1:1 ratio of Au:thiolate) in the shell, and the specific structure of Au GSH clusters is vividly depicted in Supplementary Fig. S2.

UV-vis absorption and steady-state emission spectra of Au GSH clusters (Fig. 1c) exhibit an absorption onset at 530 nm with a shoulder around 400 nm and an emission maximum at 605 nm. Notably, absorption features of Au GSH clusters are different from that of Au_25_-glutathione clusters synthesized by Tatsuma and Tsukuda’s groups (Au_25_-glutathione clusters have also been synthesized as Au_25_(SG)_18_ clusters and will be discussed later)^39^,^40^. This absorption variation highlights the difference in the highest occupied molecular orbital-lowest unoccupied molecular orbital (HOMO-LUMO) transitions of two types of Au clusters, while our synthesized Au GSH clusters are known to be a polydispersed mixture of different sizes with more than 29 Au atoms^6^. Emissive properties clearly differentiate the ultra-small Au GSH clusters from larger plasmonic Au nanoparticles (NPs). The large Stokes shift in the emission where the absorption band shoulder appears at around 400 nm and the emission maximum is seen at 605 nm, is consistent with the excited state being a ligand−metal charge-transfer type^41^,^42^. The excitation spectrum (Fig. 1c–iii) shows a maximum at 400 nm, which coincides well with the absorption shoulder observed in the absorption spectrum (Fig. 1c–i). As displayed in Supplementary Fig. S3, emission intensities of Au GSH clusters at around 605 nm are also consistent with excitation spectrum where the shape of the photoluminescence (PL) spectra is independent of the excitation wavelength.

### Characterization of Au GSH Clusters-TiO_2_ Composites

**Figure 2:**
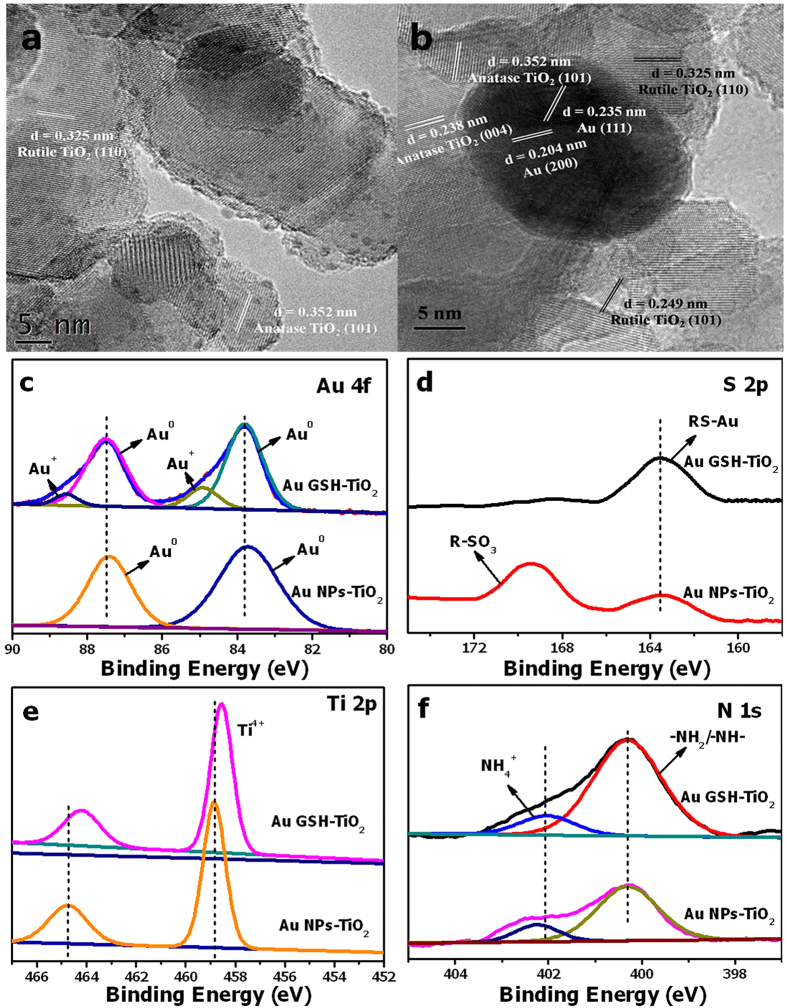
Morphology and charge states variation Au GSH clusters-TiO2 composites. HRTEM images of Au GSH clusters-TiO_2_ composites before (**a**) and after (**b**) the simulated solar light photo-irradiation and high-resolution XPS spectra of Au 4f (**c**), S 2p (**d**), Ti 2p (**e**) and N 1s (**f**) for Au GSH clusters-TiO_2_ composites before and after the simulated solar light photo-irradiation.

White TiO_2_ powders (Degussa P25) turn to light yellow after soaking the TiO_2_ in the Au GSH clusters solution, indicating the successful attachment of Au GSH clusters onto the surface of TiO_2_ nanoparticles (Supplementary Fig. S4). The absorption spectrum variation of TiO_2_ before and after soaking in the Au GSH clusters solution provides another evidence for the loading of the Au GSH clusters sensitizer on the TiO_2_ surface. As shown in Supplementary Fig. S5, the Au GSH clusters-TiO_2_ composites show step-like behavior in the absorption characteristics at around 400 nm, which is similar to that of aqueous Au GSH clusters, while TiO_2_ exhibits a narrow absorption range. Morphologies of Au GSH clusters-TiO_2_ composites have been examined by the TEM analysis as shown in Fig. 2 and Supplementary Fig. S6. Uniformly distributed Au GSH clusters with sizes in the range of 1.36 ± 0.55 nm are observed on the TiO_2_ surface, faithfully confirming that Au GSH clusters are successfully loaded on TiO_2_ nanoparticles. The spacing of the distinct lattice fringe in the HRTEM image (Fig. 2a) of the samples is determined to be 0.352 nm and 0.325 nm, corresponding to the (101) and (110) crystal plane of anatase and rutile TiO_2_, respectively, while the crystal lattice of Au GSH cluster is not observed.

To initially investigate the photo-stability of the Au GSH clusters at the Au/TiO_2_ interface, Au GSH clusters-TiO_2_ composites were exposed to the simulated solar light photo-irradiation in the air under ambient conditions. The light yellow Au GSH clusters-TiO_2_ composites turn to purple after the simulated solar light photo-irradiation for 6 h (Supplementary Fig. S7), which indicates the aggregation of ultra-small Au GSH clusters to larger metallic Au NPs at the Au/TiO_2_ interface during the light illumination. This growth of ultra-small Au GSH clusters to larger metallic Au NPs after the photo-irradiation has been confirmed by the TEM analysis (Fig. 2 and Supplementary Fig. S8). Fig. 2b exhibits the aggregated Au NPs with the size at around 15 nm over the TiO_2_ surface. In addition to the observed lattice fringe spacing of TiO_2_, the lattice fringe spacings at 0.235 nm and 0.204 nm, corresponding to the (111) crystal plane and (200) crystal plane of Au, respectively, are also evident after the photo-irradiation. This phenomenon clearly differentiates the aggregated Au NPs from Au GSH clusters whose crystal lattice is hardly observed as mentioned above. Additionally, the large Au NPs can even be seen in the SEM image (Supplementary Fig. S9). The phase composition and crystallinity variation of Au GSH clusters-TiO_2_ composites before/after the photo-irradiation have been characterized by X-ray diffraction (XRD). However, the Au diffraction peaks are not observed due to the low loading amount of Au GSH clusters and their weak intensities (Supplementary Fig. S10).

### Charge States Variation of Au GSH Clusters-TiO_2_ Composites after the Photo-irradiation

The composition and chemical valence states of the samples before and after the photo-irradiation have been probed by X-ray photoelectron spectroscopy (XPS). Survey XPS spectrum evidences the presence of Au 4f, S 2p, N 1s, C 1s, O 1s and Ti 2p elements in the Au GSH clusters-TiO_2_ composites (Supplementary Fig. S11). Fig. 2c shows two doublet 4f peaks in the high-resolution spectrum of Au 4f for Au GSH clusters-TiO_2_ composites, suggesting that there are two different elemental chemical states of Au species, which consists with the Au 4f spectrum for solid Au GSH clusters (Supplementary Fig. S12). The peaks with binding energies (BEs) of 87.50 eV and 83.80 eV are ascribed to Au 4f_5/2_ and Au 4f_7/2_ of metallic Au (Au^0^), respectively, and the other doublet peaks at 88.55 eV and 84.90 eV are assigned to Au 4f_5/2_ and Au 4f_7/2_ of Au^+^, respectively^27^,^43^,^44^,^45^. As mentioned above, the synthesized Au GSH clusters are demonstrated to possess Au(0)@Au(I)-thiolate core-shell nanostructures^26^,^38^. The XPS results provide persuasive evidence to support this statement. After the photo-irradiation, Au^+^ peaks disappeared, which further evidences the photo-induced transformation of Au GSH clusters to Au NPs. Moreover, the intensity of S 2p_3/2_ peak at 163.2 eV, corresponding to the ligand features (RS–Au) in Au GSH clusters becomes weaker after the photo-irradiation, while a new peak at 169.2 eV, matching the BE of R–SO_3_ species is observed, suggesting that the sulfur-containing ligands undergo an oxidation process during the photo-irradiation (Fig. 2d)^45^,^46^,^47^,^48^. The BE shift for Ti 2p_3/2_ and decreased N 1s peaks after the photo-irradiation also provide evidences on the organic ligands oxidation process (Fig. 2e,f). Fourier transformed infrared spectroscopy (FTIR) and Raman spectra further confirm the oxidation of sulfur-containing ligands during the photo-induced Au GSH clusters transformation process. As shown in Supplementary Fig. S13, 1628 cm^−1^ peak corresponding to stretching vibration modes of -COOH groups from GSH ligands and 1424 cm^−1^ peak for CH_2_–S methylene scissoring (δ) exist in the FTIR spectrum of Au GSH clusters-TiO_2_ composites. In contrast, for Au NPs-TiO_2_ composites, new strong peak of thiosulfonate at 1340 cm^−1^ and weak peaks of R-SO_3_ at 1179 cm^−1^ and 1042 cm^−1^ are observed after the photo-irradiation, which confirms the oxidative damage of GSH ligands during the photo-irradiation^45^. In addition, the decreased S-S stretching band in Raman spectra indirectly supports the ligands oxidation process (Supplementary Fig. S14)^49^. Since the primary role of glutathione ligands lies in stabilizing Au GSH clusters against aggregation, we postulate that the photo-induced transformation of Au GSH clusters to Au NPs originates from the oxidative attack of glutathione ligands by photo-generated holes and derivative active oxidative species (such as hydroxyl radicals) resulting from the band-gap photo-excited TiO_2_ under the simulated solar light illumination.

### Optical Absorption Variation of Au GSH Clusters-TiO_2_ Composites after the Photo-irradiation

**Figure 3:**
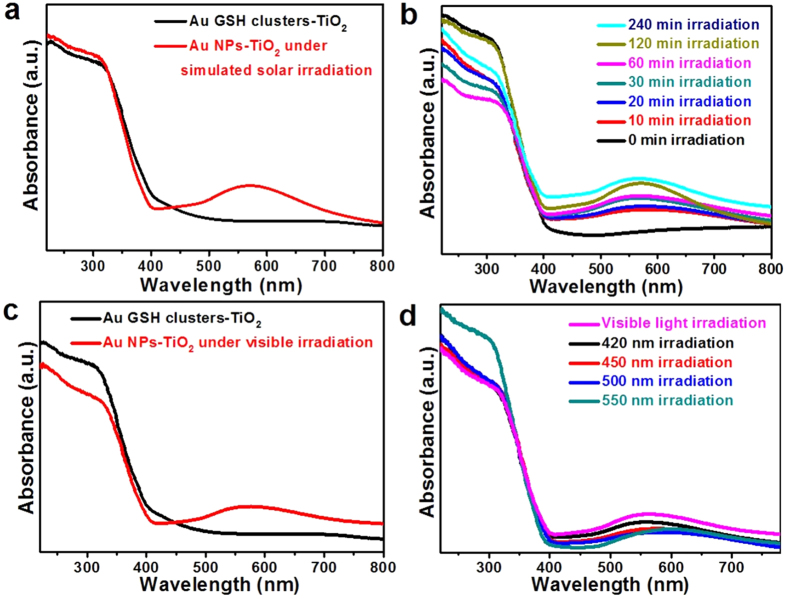
UV-vis diffuse reflectance spectra (DRS) of Au GSH clusters-TiO2 composites. Before and after the simulated solar light irradiation with 6 h (**a**) and with different irradiation times (**b**) before and after the visible light irradiation with 6 h (**c**) and with different excitation wavelength over 6 h (**d**).

Structural transformation from ultra-small molecular-like Au GSH clusters to larger metallic Au NPs during the photo-irradiation leads to an optical absorption variation. Fig. 3a shows the UV-vis diffuse reflectance spectra (DRS) of Au GSH clusters-TiO_2_ composites before/after the simulated solar light illumination. The surface plasmon resonance (SPR) peak at around 550 nm further confirms the transformation of Au GSH cluster to plasmonic Au NPs. In addition, the increased SPR intensity with the prolonged photo-irradiation time suggests that the optical properties of Au GSH clusters-TiO_2_ composites can be tuned by controlling the reaction conditions (Fig. 3b). Interestingly, the Au GSH clusters transformation is also observed under the visible light photo-irradiation as evidenced by Fig. 3c,d. While exposing the Au GSH clusters-TiO_2_ composites to the visible light illumination with wavelength from 420 nm to 550 nm, typical SPR feature of Au NPs can be also observed, even though the TiO_2_ cannot be band-gap photo-excited under the visible light photo-irradiation.

It is noteworthy that the SPR intensity of Au GSH clusters-TiO_2_ composites under the simulated solar light photo-irradiation with 6 h is obviously higher than that under the visible light photo-irradiation. As mentioned above, we hypothesize that the photo-induced transformation of Au GSH clusters should be induced by the oxidative attack of glutathione ligands. This as-observed SPR intensity difference is corresponding well with our hypothesis. During the visible light photo-irradiation, Au GSH clusters with a distinct HOMO-LUMO gap which act like a semiconductor quantum dots are photo-excited^50^,^51^, the formed active oxygen species (e.g., superoxide and hydroxyl radicals) resulting from the reactions between electron and oxygen or adsorbed water molecules^1^,^2^,^3^, would contribute to the attacks of glutathione ligands, thus leading to the Au GSH clusters aggregation. While under simulated solar light illumination, both TiO_2_ and Au GSH clusters are able to be photo-excited to generate stronger oxidative holes and derivative active oxygen species for more efficiently attacking Au GSH clusters^27^,^28^, thus resulting in the higher SPR intensity of Au NPs as shown in Fig. 3a,c.

### Photoelectrochemical Performances Variation of Au GSH Clusters-TiO_2_ Composites after the Photo-irradiation

**Figure 4:**
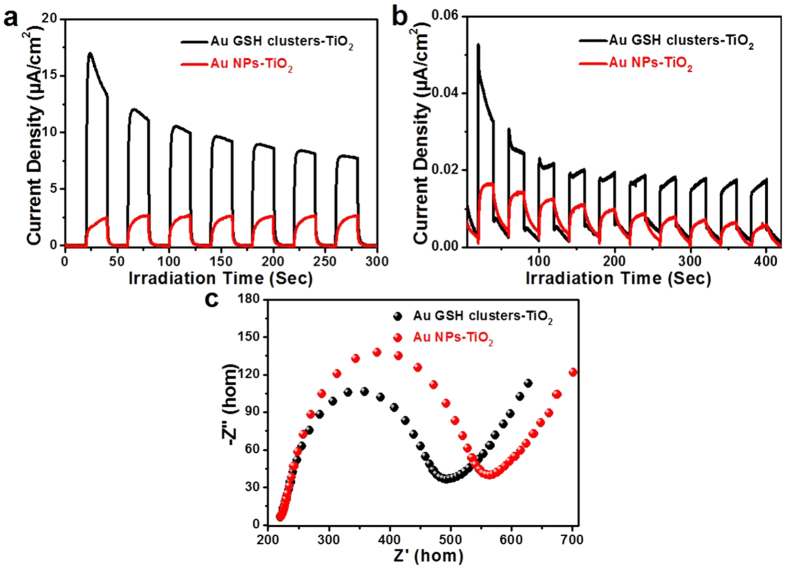
Photoelectrochemical measurements. Transient photocurrent responses of Au GSH clusters-TiO_2_ and Au NPs-TiO_2_ composites in a 0.2 M Na_2_SO_4_ (pH = 6.8) aqueous solution with zero bias versus Ag/AgCl under the simulated solar light photo-irradiation (**a**) and the visible light photo-irradiation (**b**) and electrochemical impedance spectroscopy (EIS) Nyquist plot of the samples in the solution with the presence of 10 mM K_3_[Fe(CN)_6_]/K_4_[Fe(CN)_6_] and 0.5 M KCl with zero bias in the dark, amplitude of the sinusoidal wave was set at 5 mV and frequency varied from 100 kHZ to 1 HZ (**c**).

The photoelectrochemical (PEC) performances variation of the Au GSH clusters-TiO_2_ composites before/after the photo-irradiation has also been investigated. Fig. 4a,b show the periodic on/off transient photocurrent response of Au GSH clusters-TiO_2_ composites before/after the photo-irradiation under the intermittent simulated solar light and visible light (λ > 420 nm) photo-irradiation, respectively. Apparently, the aggregated Au NPs lead to the decreased photocurrent density of Au NPs-TiO_2_ composites as compared to that of Au GSH clusters-TiO_2_, which verifies the substantial photosensitization effect of Au GSH clusters on the PEC performances of hybrid nanostructured Au GSH clusters-TiO_2_ composites in the range of ultraviolet to visible light. It is generally recognized that photocurrent is produced due to the diffusion of photo-generated electrons to the back contact and, meanwhile, the capture of photo-generated holes by electron-donor in the electrolyte^52^. Therefore, the superior photocurrent density of Au GSH clusters-TiO_2_ composites suggests more efficient separation and migration of photo-excited electron-hole charge carriers than Au NPs-TiO_2_ composites. Electrochemical impedance spectroscopy (EIS) has also been utilized to further explore the origin accounting for the PEC performances variation of Au GSH clusters-TiO_2_ composites before/after the photo-irradiation^53^,^54^. Nyquist plots in Fig. 4c show that Au GSH clusters-TiO_2_ composites have depressed semicircles at high frequencies as compared to Au NPs-TiO_2_ composites in the dark, suggesting the higher migration efficiency of photo-generated charge carriers of Au GSH clusters-TiO_2_ composites^55^,^56^. The PEC measurements support the rich optical and electrical properties of Au GSH clusters^57^,^58^.

### Photocatalytic Performances of Au GSH Clusters-TiO_2_ and Au NPs-TiO_2_ Composites

**Figure 5:**
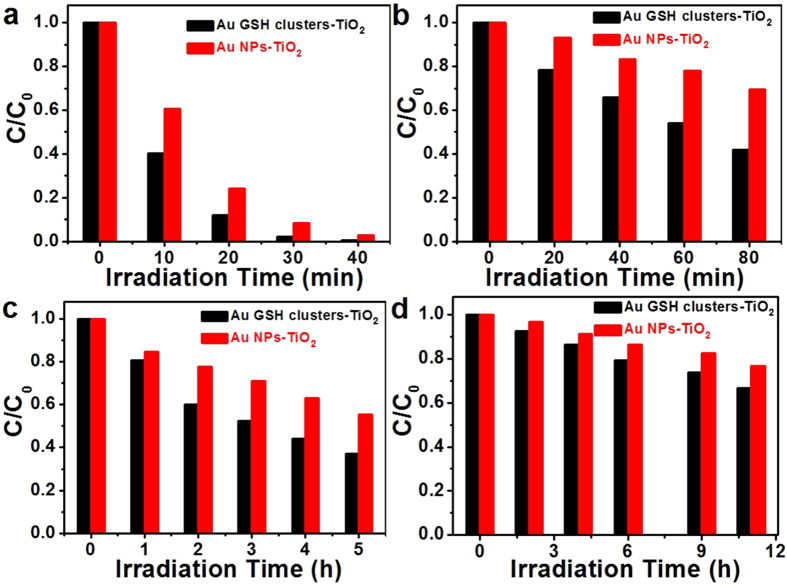
Photocatalytic performances. Photo-degradation of RhB over Au GSH clusters-TiO_2_ and Au NPs-TiO_2_ composites under the simulated solar light irradiation (**a**) and visible light irradiation (λ > 420 nm) (**b**) and photocatalytic reduction of Cr (VI) to Cr (III) over the samples under the visible light irradiation (λ > 420 nm) (**c**) and incident wavelength centered at 550 nm (**d**).

The photo-induced oxidative transformation of ultra-small Au GSH clusters to larger Au NPs leads to the altered optical and PEC features, which will directly influence the photocatalytic performances of Au GSH clusters-TiO_2_ composites before/after the photo-irradiation. Photo-degradation of organic dye pollutant has been employed as a model reaction to probe the influence of Au GSH clusters aggregation on the photocatalytic performance of Au GSH clusters-TiO_2_ composites under both the simulated solar light and visible light irradiation. As shown in Fig. 5, Au NPs-TiO_2_ composites exhibit decreased photo-activities as compared to Au GSH clusters-TiO_2_ composites under both the simulated solar light and visible light illumination. Notably, TiO_2_ cannot be band-gap photo-excited under the visible light irradiation, which suggests different photocatalytic mechanism for Au GSH clusters-TiO_2_ composites before/after the treatment of photo-irradiation. The transformation of molecular-like Au GSH clusters to plasmonic Au NPs leads to a SPR effect for driving the photocatalytic reactions. The SPR effect has been proven by photocatalytic reduction of Cr (VI) to Cr (III), which can rule out the possible dye-sensitized effects in photo-degradation of RhB. Under both the visible light irradiation and incident wavelength centered at 550 nm, Au NPs-TiO_2_ composites are photocatalytically active toward Cr (VI) reduction as displayed in Fig. 5c,d. Similar to the photo-degradation of RhB, Au GSH clusters-TiO_2_ composites still show enhanced activity as compared to that of Au NPs-TiO_2_ composites under the photo-irradiation.

## Discussion

### Formation Mechanism of Au NPs from Au GSH Clusters during the Photo-irradiation

**Figure 6:**
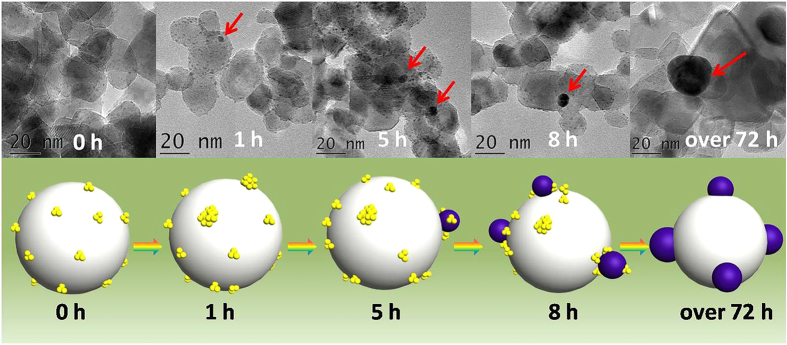
Schematic illustration depicting the formation mechanism of Au NPs by Au GSH clusters aggregation. TEM images and corresponding model illustrations of Au GSH clusters-TiO_2_ composites after the different visible light photo-irradiation time with using a 150 W Xenon lamp.

As mentioned above, we have demonstrated that the Au GSH cluster transformation is accompanied with an organic ligands oxidation process and hypothesized that the photo-induced transformation of Au GSH clusters originates from the oxidative attack of glutathione ligands. However, the detailed formation mechanism of Au NPs from Au GSH cluster during the photo-irradiation has not been clearly understood, which is important for understanding the photocatalytic mechanism of Au GSH clusters-TiO_2_ composites before/after the treatment of photo-irradiation. Therefore, TEM analysis of Au GSH clusters-TiO_2_ composites during the different photo-irradiation time has been performed to investigate the photo-induced Au GSH clusters transformation process. Due to the fast transformation of Au GSH clusters under the simulated solar light photo-irradiation in 300 W Xenon lamp, we have utilized a 150 W Xenon lamp with visible light irradiation to study the Au GSH clusters transformation process. Clearly, color changes of the samples are observed for different light illumination time (Supplementary Fig. S15), which corresponds well with the particle size variation of Au GSH clusters. Light yellow Au GSH clusters-TiO_2_ composites turn to light gray after 1 h photo-irradiation, corresponding to the slightly aggregation of Au GSH clusters with few small Au NPs at around 3 nm as shown in Fig. 6. With prolonging the photo-irradiation time to 5 h and 8 h, the sample turns to light purple, corresponding to the average particle growth to 7 nm and 10 nm, respectively. After 72 h photo-irradiation, 15 nm Au NPs are observed while Au GSH clusters almost disappear. Notably, as compared to small Au NPs observed in the case of 1 h photo-irradiation, less Au GSH clusters exist around large Au NPs in the case of 8 h photo-irradiation, which indicates a diffusion/aggregation mechanism for Au NPs formation under light illumination. Therefore, it is reasonable to believe that the photo-induced oxidative attack of glutathione ligands contributes to the easier lateral diffusion of Au GSH clusters and thus the diffusion/aggregation process Au GSH clusters.

Controlled photocatalytic reduction of Cr (VI) also provides indirect evidence for the transformative aggregation process of Au GSH clusters under light illumination conditions. As shown in Supplementary Fig. S16, with adding hole scavengers under N_2_ atmosphere, the reaction substrate Cr (VI) is almost completely reduced after 2 h visible light irradiation over Au GSH clusters-TiO_2_ composites. In contrast, without adding hole scavengers in air, only 52% of Cr (VI) is reduced after 4 h visible light irradiation over Au GSH clusters-TiO_2_ composites. For the former case, light gray samples corresponding to slightly Au GSH clusters aggregation are observed during anaerobic atmosphere with adding hole scavengers, whereas for the latter case without hole scavengers in air, the purple samples with typical SPR features of Au NPs are observed (Supplementary Fig. S17). These suggest that the Au GSH clusters aggregation can be efficiently inhibited if the oxidative attacking of Au GSH clusters is suppressed by the control of reaction conditions.

A series of controlled experiments further confirm the photo-induced oxidative aggregation of Au GSH clusters as listed in Supplementary Table S1. Apparently, both the photo-irradiation and oxidative atmosphere are necessary for the Au GSH clusters transformation process. Adding sacrificial agents in oxidative atmosphere cannot efficiently inhibit the aggregation of Au GSH clusters during the photo-irradiation, which might be due to the trapping of photo-generated electrons by molecular oxygen to form superoxide radicals for attacking the Au GSH clusters^53^,^59^. This can be verified by the suppressed Au GSH clusters aggregation in the anaerobic atmosphere with adding sacrificial agents under the photo-irradiation as displayed in Supplementary Fig. S18. After the simulated solar light photo-irradiation in vacuum for 3 h, the average particle size of Au GSH clusters grows from 1.36 to 1.57 nm. With further prolonging the photo-irradiation time to 18 h, few Au NPs around 3 nm are observed while most Au GSH clusters maintain the size under 2 nm (Supplementary Fig. S18c). However, in the anaerobic atmosphere without adding sacrificial agents, Au GSH clusters tend to aggregate under the simulated solar light irradiation; however they show the inhibited aggregation under the visible light illumination. This suggests the weak oxidative capacity of photo-generated holes from Au GSH clusters while the photo-generated holes and derivative active oxygen species from band-gap photo-excited TiO_2_ should play a dominant role for the Au GSH clusters transformation in the anaerobic atmosphere. Our controlled experiments provide more evidences on the photo-induced oxidative aggregation of Au GSH clusters to Au NPs over the TiO_2_ surface, which are consistent with the above XPS, FTIR and Raman results. In addition, this photo-induced aggregation process can be efficiently inhibited by controlling the condition parameters, thereby improving the photo-stability of Au GSH clusters-TiO_2_ composites.

Electron spin resonance (ESR) analysis helps us confirm distinct photo-induced oxidation mechanism for Au GSH clusters aggregation under the different light photo-irradiations. As shown in Supplementary Fig. S19a, both hydroxyl radical and superoxide radical signals are detectable over Au GSH clusters-TiO_2_ composites under the simulated solar light photo-irradiation for which both TiO_2_ and Au GSH clusters are able to be photo-excited. These radicals with the well-known strong oxidative capacity^1^,^2^,^3^ would contribute to the oxidative attack on Au GSH clusters. In contrast, under the visible light illumination, negligible hydroxyl radical signal but strong superoxide radical signal of Au GSH clusters-TiO_2_ composites are observed (Supplementary Fig. S19b). Since TiO_2_ cannot be band-gap photo-excited under visible light irradiation, photo-generated electrons from excited Au GSH clusters are trapped by molecular oxygen to form the superoxide radicals^1^,^2^,^3^ which primarily drives the oxidative transformation of Au GSH clusters to Au NPs. As proved by the above controlled experiments, the photo-generated holes from photo-excited Au GSH clusters exhibit negligible influence on the oxidative aggregation of Au GSH clusters. Therefore, superoxide radicals would play a dominant role in the oxidative aggregation process of Au GSH clusters during the visible light photo-irradiation.

To further confirm our inference about photo-induced oxidation mechanism for Au GSH clusters aggregation and achieve a generally valid conclusion, the mostly studied Au_25_(SG)_18_ clusters which are well-known as the most stable one among the thiolated clusters of this class, have been utilized to modify the TiO_2_ powders to form Au_25_(SG)_18_-TiO_2_ composites and their photo-stability has been investigated under the similar conditions as those for the polydispersed Au GSH clusters. As shown in Supplementary Fig. S20, the different optical absorption between Au_25_(SG)_18_ clusters and Au GSH clusters verifies the two different types of Au clusters. During the 4 h simulated solar light photo-irradiation, no obvious color change of light pink Au_25_(SG)_18_-TiO_2_ composites is observed, which indicates the stability of Au_25_(SG)_18_ clusters at Au/TiO_2_ interface in a short-term. However, it turns to purple with prolonging irradiation time to 6 h, suggesting the photo-induced oxidative aggregation of Au_25_(SG)_18_ clusters to Au NPs (Supplementary Fig. S21). The decreased photocatalytic performance for RhB photo-degradation of Au_25_(SG)_18_-TiO_2_ composites after the simulated solar light photo-irradiation (e.g., Au NPs-TiO_2_ composites) also confirms this photo-induced transformation (Supplementary Fig. S22). Characterization results of Au_25_(SG)_18_-TiO_2_ composites before/after the photo-irradiation show the similar trend as compared to Au GSH clusters-TiO_2_ composites. XPS, FTIR and Raman analysis provides sufficient evidences on the oxidation of sulfur-containing ligands during the photo-irradiation as shown in Supplementary Fig. S23. These results convincingly have strengthened our proposed photo-induced oxidation mechanism for the organic ligands protected-Au clusters at the Au/semiconductor interface.

### Photocatalytic Mechanism of Au GSH Clusters-TiO_2_ and Au NPs-TiO_2_ Composites

**Figure 7:**
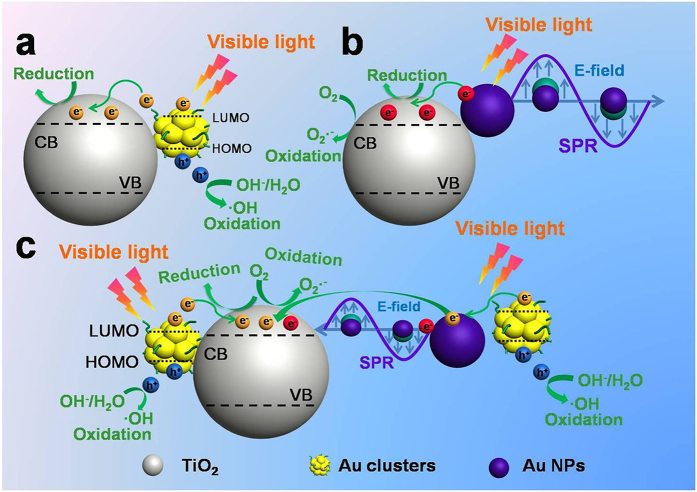
Schematic illustration depicting the photocatalytic mechanisms. Ideal Au GSH clusters-TiO_2_ composites (**a**), Au NPs-TiO_2_ composites (**b**) and practical Au GSH clusters-TiO_2_ composites (**c**) under the visible light irradiation (λ > 420 nm).

Different intrinsic feature between Au GSH clusters and Au NPs caused by photo-irradiation leads to the distinct photocatalytic performances and reaction mechanism of Au GSH clusters-TiO_2_ and Au NPs-TiO_2_ composites. According to the Kohn-Sham molecular orbitals (MOs) theory, various electron transitions from the occupied levels to the unoccupied levels can be triggered over Au GSH clusters under the light irradiation, thereby imparting Au GSH clusters with a distinct HOMO-LUMO gap which acts like a semiconductor with a small band-gap^50^,^51^. Therefore, Au GSH clusters are able to serve as a visible light photosensitizer, which can be photo-excited to generate electron-hole pairs owing to its favorable band-gap, as proved by previous studies^27^,^28^. Because of the more negative LUMO potential of Au GSH clusters than the conduction band (CB) edge of TiO_2_ and the intimate interfacial contact between them, photo-generated electrons from Au GSH clusters can be readily injected into the CB of TiO_2_ (Supplementary Fig. S24)^27^,^38^,^60^. Thus, photo-generated electron-hole charge carriers over Au GSH clusters-TiO_2_ can be efficiently separated, and consequently drive the photocatalytic redox reactions together with their induced highly active oxygen species (Fig. 7a). Photoluminescence (PL) spectra provide evidence for the prolonged lifetime of the photo-generated charge carriers^61^. As mirrored in Supplementary Fig. S25, PL intensity of aqueous Au GSH clusters is gradually quenched with increasing amount of TiO_2_ with excitation wavelength at 420 nm, thus suggesting the efficient separation of photo-generated charge carriers over Au GSH clusters. On the other hand, photo-induced oxidative aggregated Au NPs with intrinsic SPR feature, can be excited under the visible light irradiation, thus generating hot electrons which can be injected into the CB edge of TiO_2_ and thereby drive the photocatalytic reactions (Fig. 7b)^62^.

Notably, light yellow Au GSH clusters-TiO_2_ composites also turn to purple during the photocatalytic degradation of RhB process under the photo-irradiation, which implies the occurrence of oxidative aggregation of Au GSH clusters to Au NPs. This variation increases the complexity of the photocatalytic mechanism of Au GSH clusters-TiO_2_ composites, which is different from Fig. 7a,b. As proved by TEM analysis in Fig. 6 and Supplementary Fig. S26, both Au GSH clusters and Au NPs are co-existed during the Au GSH clusters transformation process, which indicates a synergistic effect between molecular-like Au GSH clusters and plasmonic Au NPs, as displayed in Fig. 7c. On one hand, photo-excited electrons from Au GSH clusters can directly transfer to the CB of TiO_2_ based on suitable band alignment between clusters and TiO_2_ (Fig. 7a). On the other hand, it is feasible for involving the first transfer of electrons from clusters to the Au NPs and then to the CB of TiO_2_ in which Au NPs act as a bridging medium to reinforce the flow of electrons^63^. In addition, the SPR induced localized oscillating electric field of Au NPs also facilitates the transport of electrons photo-generated over Au GSH clusters^8^. Furthermore, plasmon excitation generated hot electrons on Au NPs can be also injected into the CB of TiO_2_, therefore increasing the density of photo-generated electrons in the whole reaction system. Controlled Cr (VI) reduction experiments provide evidences to such proposed synergistic photocatalytic effect. As displayed in Supplementary Fig. S27, Au GSH clusters-TiO_2_ composites after 1 h visible light photo-irradiation treatment lead to the simultaneous existence of both Au GSH clusters and Au NPs, which show the higher photo-activity than both original Au GSH clusters-TiO_2_ composites and Au NPs-TiO_2_ composites. This result is consistent with the proposed synergistic photocatalytic mechanism. However, it should be emphasized that photo-stability effects of the samples during the photocatalytic process on the photocatalytic mechanism are not taken into account in the above experiments. In fact, with prolonging the photo-irradiation time, the gradual aggregation of Au GSH clusters to Au NPs leads to the varying compositions of Au GSH clusters-TiO_2_ composites, which further increases the complexity of Au GSH clusters-TiO_2_-based photocatalysis with a dynamically mutative photocatalytic mechanism during the photocatalytic reactions.

As discussed above, the photo-induced oxidative aggregation of organic ligands protected-Au clusters to Au NPs involves the oxidation of glutathione ligands, growth of Au clusters and different photocatalytic mechanisms, which increase the complexity of organic ligands protected-Au clusters-based photocatalysis. In this case, it is rather difficult to draw a conclusion on the genuine advantage of the ultra-small molecular-like Au GSH clusters as cocatalysts over larger metallic plasmonic Au NPs in promoting the photocatalysis. Therefore, further systematic studies on the influence of the clusters transformation on the photocatalytic activity and mechanism of metal cluster-semiconductor composites should be unambiguously established.

In summary, photo-induced transformation of ultra-small Au GSH clusters to larger metallic Au NPs at the Au/TiO_2_ interface has been observed under the photo-irradiation. Such a transformation has been demonstrated to be a photocatalytic oxidative process with diffusion/aggregation mechanism. The variation of ultra-small Au GSH clusters from molecular-like photosensitizer with small band-gap to metallic plasmonic Au NPs during the photo-irradiation, results in the altered optical and photoelectrochemical properties and distinct photocatalytic performances with different mechanism. In addition, it has been found that the photo-induced transformation of Au GSH clusters can be triggered by both photo-excited TiO_2_ under the simulated solar light irradiation and excited Au GSH clusters themselves under the visible light illumination.

A positive outcome of the present study is that the photo-induced transformation of Au GSH clusters can be inhibited to some extent by controlling the reaction conditions. Nonetheless, the long-term photo-stability of Au clusters-semiconductor composites needs intelligent structural design. In this regard, encapsulation of Au clusters with a stable semiconductor shell forming core-shell nanostructure composites holds promising potential for preventing the aggregation of ultra-small clusters during the photo-irradiation and provides a platform to study the unique optical and electrical properties of metal clusters. It is hoped that this work could raise the significant concern on the judicious use of metal clusters in diverse photocatalytic applications and promote the rational structural design and reaction condition control toward achieving metal clusters-semiconductor composite photocatalyst with long-term photo-stability for target applications.

## Methods

### Chemicals

Gold(III) chloride trihydrate (HAuCl_4_·3H_2_O), acetonitrile (HPLC grade), sodium hydroxide (NaOH) and hydrochloric acid (HCl) were obtained from Sinopharm Chemical Reagent Co., Ltd. (Shanghai, China), Degussa P25 TiO_2_ nanoparticle powders (EVONIK Industries), L-glutathione reduced (Sigma-Aldrich). All materials were analytical grade and used as received without further purification. Deionized water used in the synthesis was from local sources.

### Synthesis of Au GSH Clusters

Synthesis of Au GSH clusters was adopted, with modification, from the literature report by Luo *et al*.^26^,^38^. In brief, 0.24 g of gold (III) chloride trihydrate was dissolved in 300 mL deionized water (DI water) at room temperature in a 500 mL round-bottom flask. Then, 0.276 g of L–glutathione was introduced to this solution under stirring. The mixture was kept stirring until a colorless solution was obtained and then heated at 70 °C in an oil bath with constant stirring. After 24 h, this flask was removed from the oil bath and allowed to cool to room temperature. The as-synthesized Au GSH clusters aqueous solution was purified by adding acetonitrile to recrystallize the cluster and then thoroughly washed with DI H_2_O and acetonitrile (1:3 in volume) for three times by centrifuging at 7800 rpm for 5 min. The purified Au GSH clusters were dried under air flow and redispersed in DI water to a desired concentration (with the assistance of few drops of 0.5 M NaOH) and finally stored in a refrigerator at 4 °C for further use.

### Synthesis of Au_25_(SG)_18_ Clusters

A mixture of glutathione-protected Au clusters was synthesized according to the literature^64^ with some modifications. Briefly, glutathione (reduced form, 1 mmol) was added to methanol (50 mL) containing HAuCl_4_·3H_2_O (0.25 mmol). Under vigorous stirring, an ice-cold NaBH_4_ aqueous solution (0.2 M, 12.5 mL) was added and aged for 1 h. The obtained precipitate was thoroughly washed with methanol and dried in vacuum at room temperature to obtain a mixture of gold clusters.

The mixture (4.9 mg) was dissolved in an aqueous solution (7 mL) containing glutathione (130.7 mg) and stirred at 55 °C under air bubbling for 6–9 h to obtain Au_25_(SG)_18_ cluster^65^. To remove excess glutathione, the obtained solution containing the Au_25_(SG)_18_ cluster was loaded into a dialysis membrane (MWCO 8000) and stirred slowly at <10 °C for 12 h. The precipitate formed during dialysis was removed with a filter (pore size, 0.2 μm). It was confirmed by polyacrylamide gel electrophoresis that the obtained solution contains no other clusters^40^.

### Preparation of Organic Ligands Protected-Au Clusters Modified TiO_2_ Composites

The Degussa P25 nanoparticle powders were added into an Au GSH clusters or Au_25_(SG)_18_ clusters solution (2 wt%, pH ≈ 4, adjusted by NaOH or HCl) and kept stirring at room temperature for 4 h. The interaction between carboxylic acid groups of the organic ligands protected-Au clusters and the TiO_2_ surface seem to facilitate the deposition at this moderately acidic pH (Supplementary Fig. S28)^40^. The organic ligands protected-Au clusters-TiO_2_ composites were then washed thoroughly with DI water and ethanol and dried in oven at 60 °C.

### Characterizations

The crystal phase properties of the samples were analyzed with a Bruker D8 Advance X-ray diffractometer (XRD) using Ni-filtered Cu Kα radiation at 40 kV and 40 mA in the 2θ ranging from 10° to 80° with a scan rate of 0.02° per second. Absorption spectra of Au GSH clusters were measured using a Varian Cary-50 UV–vis spectrophotometer. The optical properties of the samples were analyzed using a UV–vis spectrophotometer (Cary-500, Varian Co.) in which BaSO_4_ was used as the background. Field-emission scanning electron microscopy (FE-SEM) was used to determine the morphology of the samples on a FEI Nova NANOSEM 230 spectrophotometer. Transmission electron microscopy (TEM) and high-resolution transmission electron microscopy (HRTEM) images were obtained using a JEOL model JEM 2010 EX instrument at an accelerating voltage of 200 kV. X-ray photoelectron spectroscopy (XPS) measurements were performed using a Thermo Scientific ESCA Lab250 spectrometer which consists of a monochromatic Al Kα as the X-ray source, a hemispherical analyzer and sample stage with multi-axial adjustability to obtain the composition on the surface of samples. The Fourier transformed infrared spectroscopy (FTIR) was performed on a Nicolet Nexus 360 FTIR spectrophotometer at a resolution of 4 cm^−1^. Raman spectroscopic measurements were performed on a Renishaw inVia Raman System 1000 with a 532 nm Nd:YAG excitation source at room temperature. The photoluminescence (PL) spectra for liquid samples were investigated on an Edinburgh FL/FS900 spectrophotometer with different excitation wavelength from 340 nm to 450 nm. Excitation spectra were recorded by monitoring emission at 605 nm. Excitation and emission spectra are normalized to the 400 nm absorption. The photocurrent measurements were carried out on a BAS Epsilon workstation with homemade three electrode quartz cells. The electrolyte was 0.2 M aqueous Na_2_SO_4_ solution (pH = 6.8) without additive. The electrochemical impedance spectroscopy (EIS) measurements were implemented on an AutoLab μAUTIII.FRA2.v electrochemical workstation (Eco Chemie, The Netherlands) with the presence of 10 mM K_3_[Fe(CN)_6_]/K_4_[Fe(CN)_6_] and 0.5 M KCl by applying an AC voltage with 5 mV amplitude in a frequency range from 1 Hz to 100 kHz under open circuit potential conditions. Electron spin resonance (ESR) signal of the radical species that are spin-trapped by 5,5-dimethyl-1-pyrroline-N-oxide (DMPO) was measured using a Bruker EPR A300 spectrometer. The irradiation source was a 300 W Xe arc lamp system with or without a UV–cut filter to cut off light with a wavelength λ < 420 nm.

### Degradation of Rhodamine B (RhB)

The photocatalytic degradation of Rhodamine B (RhB) was performed in a self-designed photochemical reactor equipped with an electromagnetic stirrer. In a 100 ml glass flask equipped with a magnetic stir bar, 10 mg of catalyst was dispersed in 40 ml of a 10 mg·L^–1^ aqueous solution of RhB. The mixture was stirred in the dark for 1 h to ensure the establishment of adsorption–desorption equilibrium between the sample and reactant. Then, the above suspension was irradiated with a 300 W Xe arc lamp (PLS-SXE 300, Beijing Perfect Light Co., Ltd.) with and without a UV–cut filter (to cut off light with a wavelength λ < 420 nm) under ambient conditions and stirring, respectively. During the process of the reaction, 4 mL of sample solution was taken from the reaction system at a certain time interval. Then, the solid photocatalyst was immediately separated from the mixed phase by centrifugation, and the remaining supernatant was analyzed on a Varian UV–vis spectrophotometer (Cary 50, Varian Co.).

### Selective Reduction of Cr (VI)

In a 100 mL glass flask equipped with a magnetic stir bar and a three-hole plug, 10 mg of photocatalyst was dispersed in 40 ml Cr (VI) suspensions (10 mg·L^−1^) with 40 mg of ammonium formate as hole scavenger. Cr (VI) suspension (10 mg·L^−1^) was prepared by dissolving K_2_Cr_2_O_7_ into deionized water. The mixed suspension was first magnetically stirred in the dark for 1 h to reach the adsorption–desorption equilibrium. Then, the above mixture was irradiated with a 300 W Xe arc lamp (PLS-SXE 300, Beijing Perfect Light Co., Ltd.) with a UV–cut filter to cut off light with a wavelength λ < 420 nm under ambient conditions and stirring. At certain time intervals, 4 ml of the mixed suspension was extracted and centrifuged to remove the photocatalyst. The filtrates were analyzed by recording UV–vis spectra of Cr (VI) using a Varian UV–vis spectrophotometer (Cary–50. Varian Co.). The whole experimental process was conducted under N_2_ bubbling at the flow rate of 80 mL·min^–1^. Controlled photoactivity experiments were performed similar to the above photocatalytic reduction of Cr (VI) except that the suspension was irradiated by a 300 W Xe arc lamp with a UV-CUT filter and a band-pass filter to make the wavelength of incident light at λ = 550 ± 15 nm.

## Additional Information

**How to cite this article**: Liu, S. and Xu, Y.-J. Photo-induced transformation process at gold clusters-semiconductor interface: Implications for the complexity of gold clusters-based photocatalysis. *Sci. Rep.* **6**, 22742; doi: 10.1038/srep22742 (2016).

## Supplementary Material

Supplementary Information
