# Supplementary material for: Photo-induced transformation process at gold clusters-semiconductor interface: Implications for the complexity of gold clusters-based photocatalysis
Source: Sci Rep. 2016 Mar 7;6:22742. doi: 10.1038/srep22742 (PMC4780007; doi:10.1038/srep22742)
Supplement: Supplementary Information [file srep22742-s1.doc]

**Supplementary Information**

**Photo-induced transformation process at gold clusters-semiconductor interface. Implications for the complexity of** **gold clusters-based photocatalysis**

*Siqi Liu^1,2^, Yi-Jun Xu^1,2,*^*

^1^ State Key Laboratory of Photocatalysis on Energy and Environment, College of Chemistry, Fuzhou University, Fuzhou, 350002, P. R. China.

^2^ College of Chemistry, New Campus, Fuzhou University, Fuzhou 350108, P. R. China

^*^To whom correspondence should be addressed. Tel. /Fax: +86 591 83779326

E-mail: yjxu@fzu.edu.cn

**Contents list**

**Figure S1.** High-resolution TEM image of Au GSH clusters aqueous solution.

**Figure S2.** The schematic illustration of nanostructures of Au GSH clusters and surface ligands.

**Figure S3.** Emission spectra of Au GSH clusters aqueous solution under different excitation wavelength.

**Figure S4.** Digital photographs of TiO_2_ before (left) and after (right) soaking in the aqueous Au GSH clusters suspension for 4 h.

**Figure S5.** UV-vis absorption spectra of Au GSH clusters in aqueous suspension, Degussa P25, and Au GSH clusters-TiO_2_ composites.

**Figure S6.** Typical TEM images of Au GSH clusters-TiO_2_ composites at different magnifications.

**Figure S7.** Digital photographs of Au GSH clusters-TiO_2_ composites before (left) and after (right) the simulated solar light photo-irradiation for 6h by using a 300 W Xenon lamp.

**Figure S8.** Typical TEM images of Au NPs-TiO_2_ composites at different magnifications.

**Figure S9.** Typical SEM image of Au NPs-TiO_2_ composites.

**Figure S10.** XRD patterns of Au GSH clusters-TiO_2_ composites, Au NPs-TiO_2_ composites and solid Au GSH clusters, in which A is the anatase phase and R is the rutile phase.

**Figure S11.** Survey XPS spectrum of Au GSH clusters-TiO_2_ composites.

**Figure S12.** Survey XPS spectrum (a) and high-resolution XPS spectra of Au 4f (b), S 2p (c) and N 1s (d) for Au GSH clusters solids.

**Figure S13.** Fourier transformed infrared (FTIR) spectra of Au GSH clusters-TiO_2_ and Au NPs-TiO_2_ composites.

**Figure S14.** Raman spectra of Au GSH clusters solids, Au GSH clusters-TiO_2_ and Au NPs-TiO_2_ composites.

**Figure S15.** Photographs of Au GSH clusters-TiO_2_ composites after the different photo-irradiation time under visible light by using a 150 W Xenon lamp.

**Figure S16.** Photocatalytic reduction of Cr (VI) to Cr (III) over Au GSH clusters-TiO_2_ composites under the visible light irradiation during different reaction conditions.

**Figure S17.** UV-vis diffuse reflectance spectra (DRS) of Au GSH clusters-TiO_2_ composites and recycled Au GSH clusters-TiO_2_ composites for photocatalytic reduction of Cr (VI) to Cr (III) under the visible light irradiation during different reaction conditions.

**Figure S18.** TEM images and size distribution histograms of Au GSH clusters of Au GSH clusters-TiO_2_ composites (a) before the photo-irradiation, (b) after the simulated solar light photo-irradiation in vacuum with 3 h and (c) 18 h by using a 300 W Xenon lamp.

**Figure S19.** ESR spectra of hydroxyl (in H_2_O) and superoxide radical (in MeOH) species trapped by DMPO over the Au GSH clusters-TiO_2_ composites under (a) simulated solar light and (b) visible light irradiation by using a 300 W lamp.

**Figure S20.** UV-vis absorption and digital photographs of Au GSH clusters and Au_25_(SG)_18_ clusters in aqueous suspension (a) and TEM image of Au_25_(SG)_18_ clusters (b).

**Figure S21.** TEM images of Au_25_(SG)_18_ clusters-TiO_2_ composites (a), Au NPs-TiO_2_ composites (b) and digital photographs of Au_25_(SG)_18_ clusters-TiO_2_ composites before/after the simulated solar light and visible light photo-irradiation by using a 300 W Xenon lamp (c).

**Figure S22.** Photo-degradation of RhB over Au_25_(SG)_18_ clusters-TiO_2_ composites and Au NPs-TiO_2_ composites under the simulated solar light irradiation (a) and visible light irradiation (b) by using a 300 W Xenon lamp.

**Figure S23.** High-resolution XPS spectra of Au 4f (a), Ti 2p (b), N 1s (c) and S 2p (d); Raman (e) and FTIR (f) spectra for Au_25_(SG)_18_ clusters-TiO_2_ composites before/after the simulated solar light photo-irradiation.

**Figure S24.** Simplified Au GSH clusters and TiO_2_ energy level diagram that was created from the data in ref 28.

**Figure S25.** Emission spectra of Au GSH clusters aqueous solution adding different amount of TiO_2_. Excitation wavelength is 420 nm (3 mL of Au GSH clusters aqueous solution).

**Figure S26.** TEM images of Au GSH clusters-TiO_2_ composites after 5 h visible light photo-irradiation treatment by using a 150 W Xenon lamp, which shows the co-existence of both Au GSH clusters and Au NPs.

**Figure S27.** Photocatalytic reduction of Cr (VI) to Cr (III) over Au GSH clusters-TiO_2_ composites, Au GSH clusters-TiO_2_ composites after 1 h visible light photo-irradiation treatment by using a 150 W Xenon lamp and Au NPs-TiO_2_ composites under the visible light irradiation by using a 300 W Xenon lamp.

**Figure S28.** Digital photographs of the synthesis of Au GSH clusters-TiO_2_ composites under different pH value.

**Table S1.** Summary of the color change results of a series of controlled experiments.


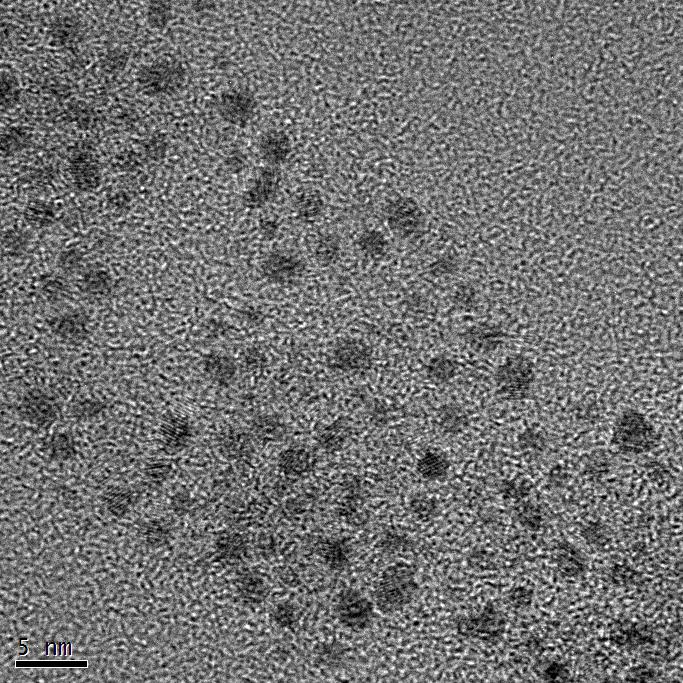


**Figure S1.** High-resolution TEM image of Au GSH clusters aqueous solution.

**Note:** The crystal lattice of Au is hardly observed in this high-resolution TEM image of Au GSH clusters aqueous solution.


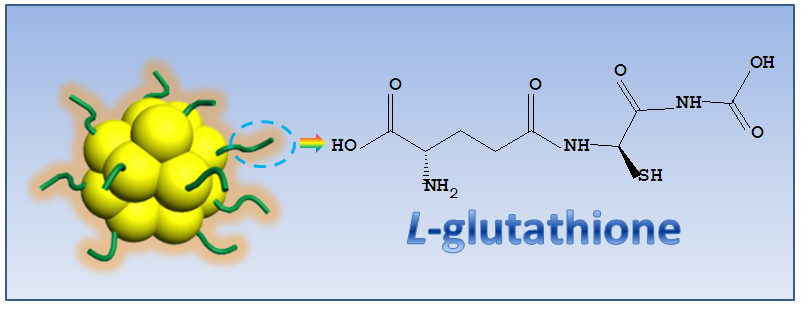


**Figure S2.** The schematic illustration of nanostructures of Au GSH clusters and surface ligands.

**Figure S3.** Emission spectra of Au GSH clusters aqueous solution under different excitation wavelength.

**Note:** The different excitation wavelengths in PL measurements are from 340 nm to 450 nm.


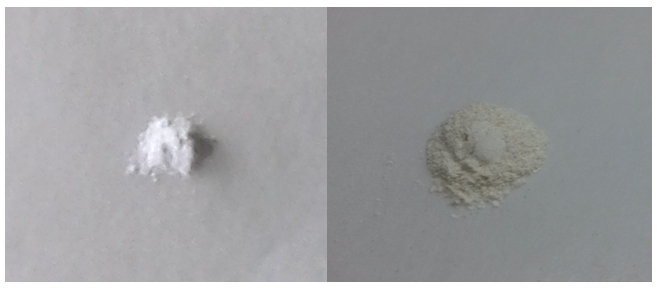


**Figure S4.** Photographs of TiO_2_ before (left) and after (right) soaking in the aqueous Au GSH clusters suspension for 4 h.

**Figure S5.** UV-vis absorption spectra of Au GSH clusters in aqueous suspension, Degussa P25, and Au GSH clusters-TiO_2_ composites.


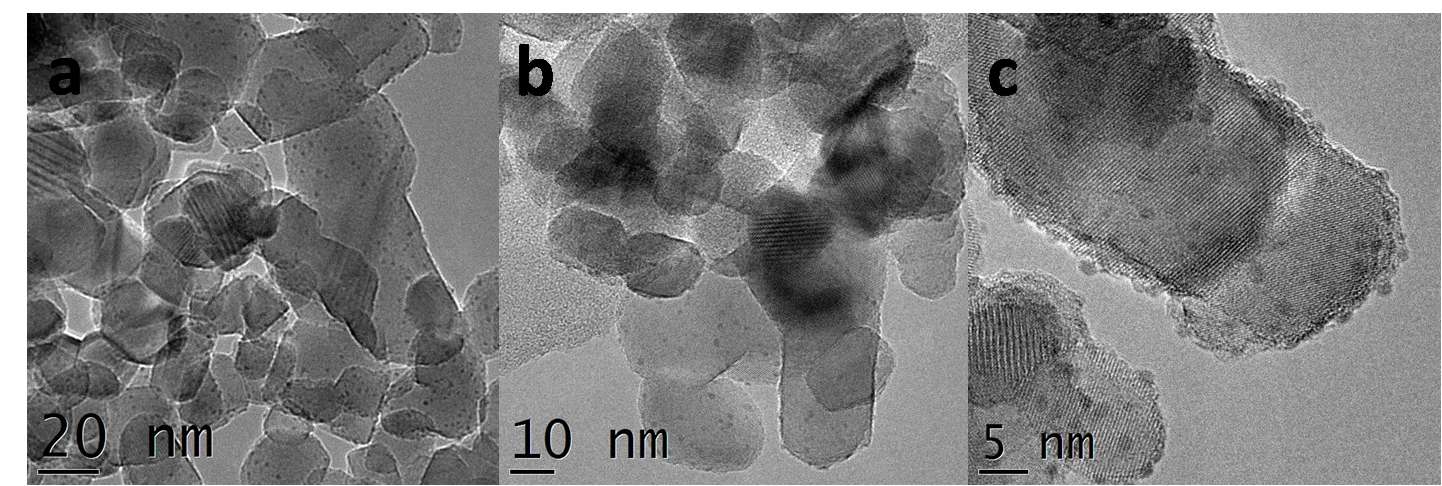


**Figure S6.** Typical TEM images of Au GSH clusters-TiO_2_ composites at different magnifications.


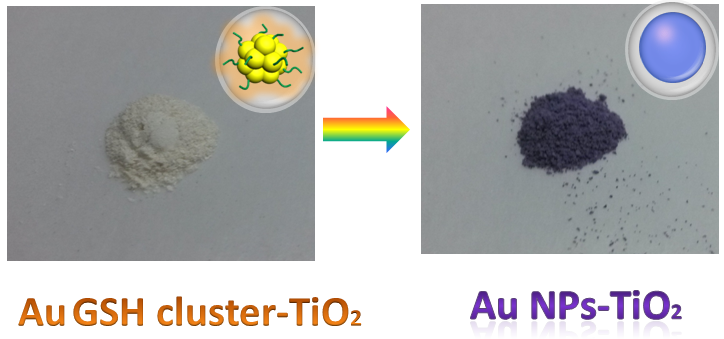


**Figure S7.** Digital photographs of Au GSH clusters-TiO_2_ composites before (left) and after (right) the simulated solar light photo-irradiation for 6h in a 300 W Xenon lamp.

**Note:** Color change from yellow to purple implies the transformation of ultra small molecular-like Au GSH clusters to larger metallic plasmonic Au NPs over TiO_2_ surface.


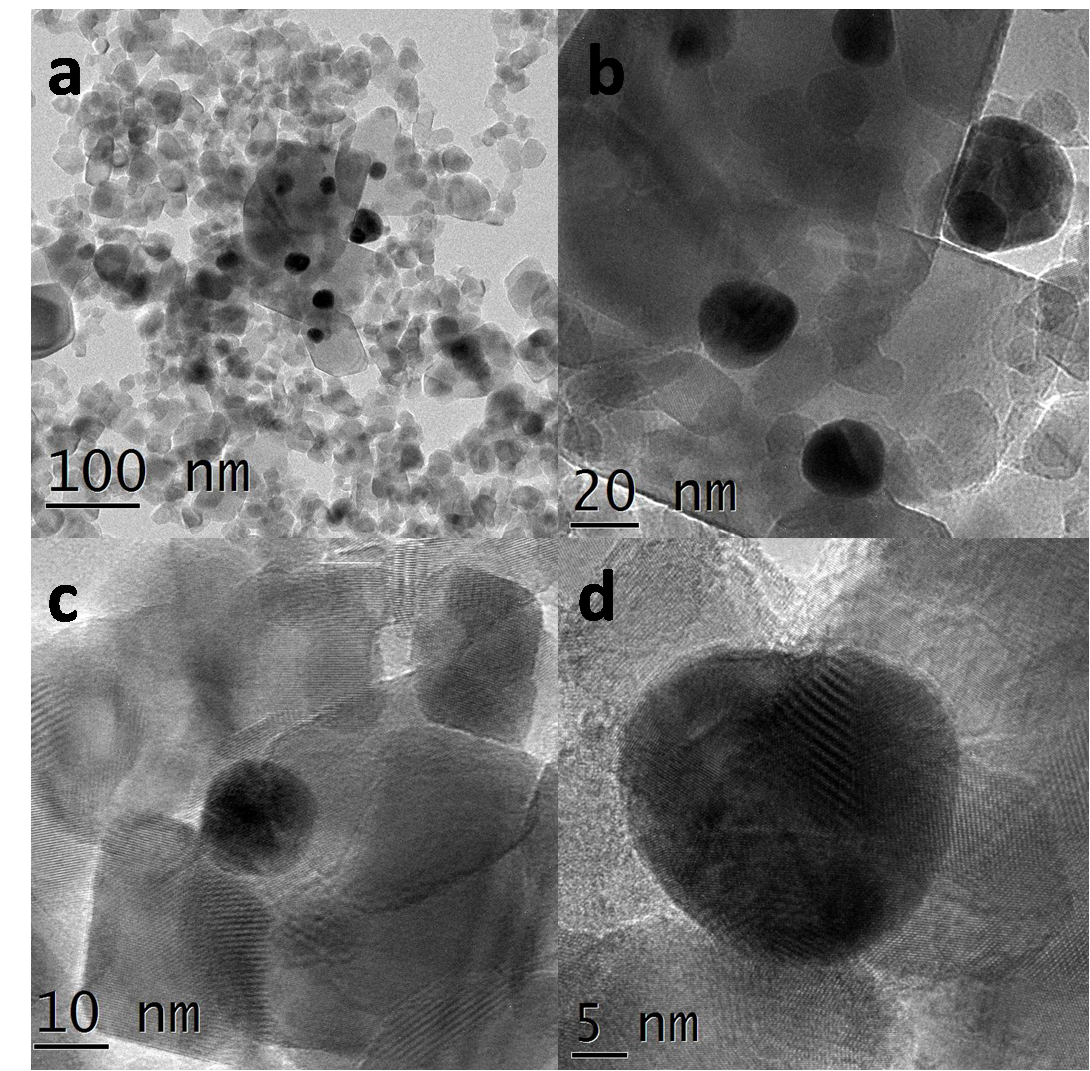


**Figure S8.** Typical TEM images of Au NPs-TiO_2_ composites at different magnifications.


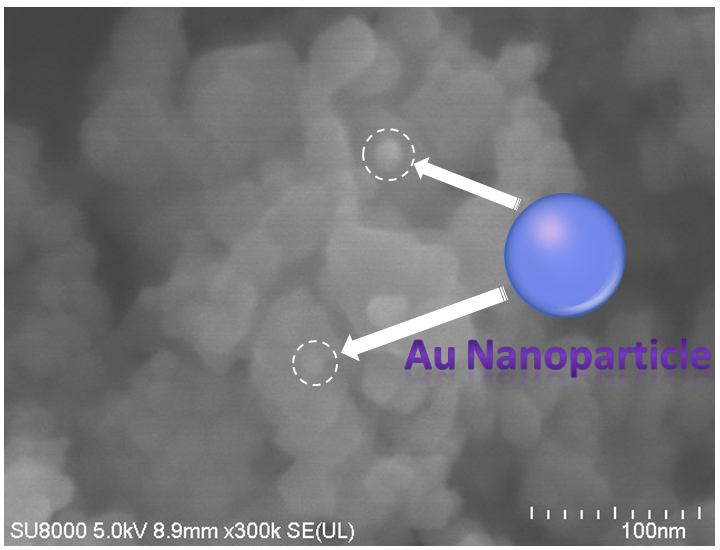


**Figure S9.** Typical SEM image of Au NPs-TiO_2_ composites.

**Figure S10.** XRD patterns of Au GSH clusters-TiO_2_ composites, Au NPs-TiO_2_ composites and solid Au GSH clusters, in which A is the anatase phase and R is the rutile phase.

**Figure S11.** Survey XPS spectrum of Au GSH clusters-TiO_2_ composites.

**Figure S12.** Survey XPS spectrum (a) and high-resolution XPS spectra of Au 4f (b), S 2p (c) and N 1s (d) for Au GSH clusters solids.

**Figure S13.** Fourier transformed infrared (FTIR) spectra of Au GSH clusters-TiO_2_ and Au NPs-TiO_2_ composites.

**Note:** 1628 cm^-1^ peak corresponds to stretching vibration modes of -COOH groups from GSH ligands; 1424 cm^-1^ peak corresponds to CH_2_−S methylene scissoring (δ) in Au GSH clusters-TiO_2_ composites. Strong 1340 cm^-1^ peak is for thiosulfonate as well as weak 1179 cm^-1^ and 1042 cm^-1^ peak for R-SO_3_ in Au NPs-TiO_2_ composites.^45^

**Figure S14.** Raman spectra of Au GSH clusters solids, Au GSH clusters-TiO_2_ and Au NPs-TiO_2_ composites.

**Note:** Five characteristic peaks at 146 (Eg), 195 (Eg), 399 (B1g), 517 (A1g + B1g) and 639 (Eg) cm^-1^ for Au GSH clusters-TiO_2_ and Au NPs-TiO_2_ composites can be assigned to TiO_2_.^63^ The peak at 490 cm^-1^ corresponds to S-S stretching.^49^

**
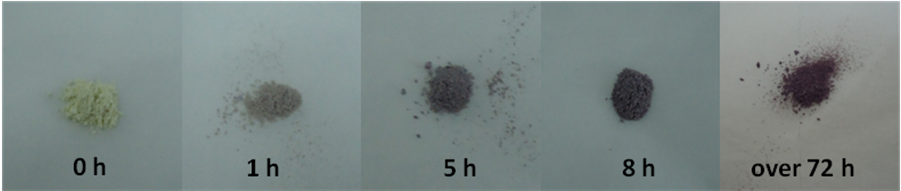
**

**Figure S15.** Photographs of Au GSH clusters-TiO_2_ composites after the different photo-irradiation time under visible light (λ > 420 nm) by using a 150 W Xenon lamp.

**Figure S16.** Photocatalytic reduction of Cr (VI) to Cr (III) over Au GSH clusters-TiO_2_ composites under the visible light irradiation (λ > 420 nm) during different reaction conditions.

**Note:** The sacrificial agent which was utilized in the photocatalytic reduction of Cr (VI) is ammonium formate.

**Figure S17.** UV-vis diffuse reflectance spectra (DRS) of Au GSH clusters-TiO_2_ composites and recycled Au GSH clusters-TiO_2_ composites for photocatalytic reduction of Cr (VI) to Cr (III) under the visible light irradiation (λ > 420 nm) during different reaction conditions.


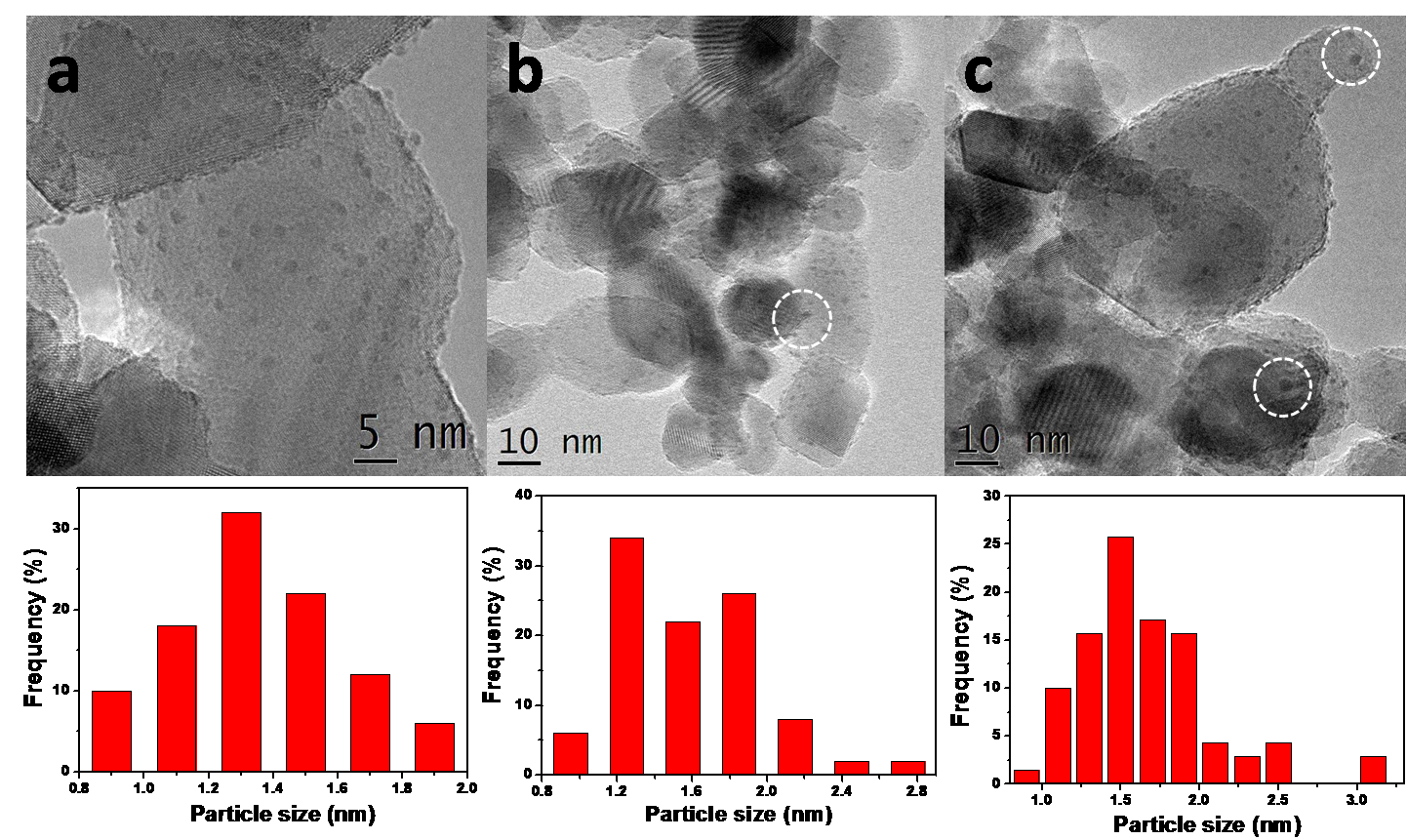


**Figure S18.** TEM images and size distribution histograms of Au GSH clusters of Au GSH clusters-TiO_2_ composites (a) before the photo-irradiation, (b) after the simulated solar light photo-irradiation in vacuum with 3 h and (c) 18 h by using a 300 W Xenon lamp and adding lactate as sacrificial agent.

**Figure S19.** ESR spectra of hydroxyl (in H_2_O) and superoxide radical (in MeOH) species trapped by DMPO over the Au GSH clusters-TiO_2_ composites under the (a) simulated solar light and (b) visible light irradiation in a 300 W Xenon lamp.

**Note:** Four peaks in H_2_O are corresponding to hydroxyl signal and six peaks in MeOH are corresponding to superoxide radical signal.^S1^


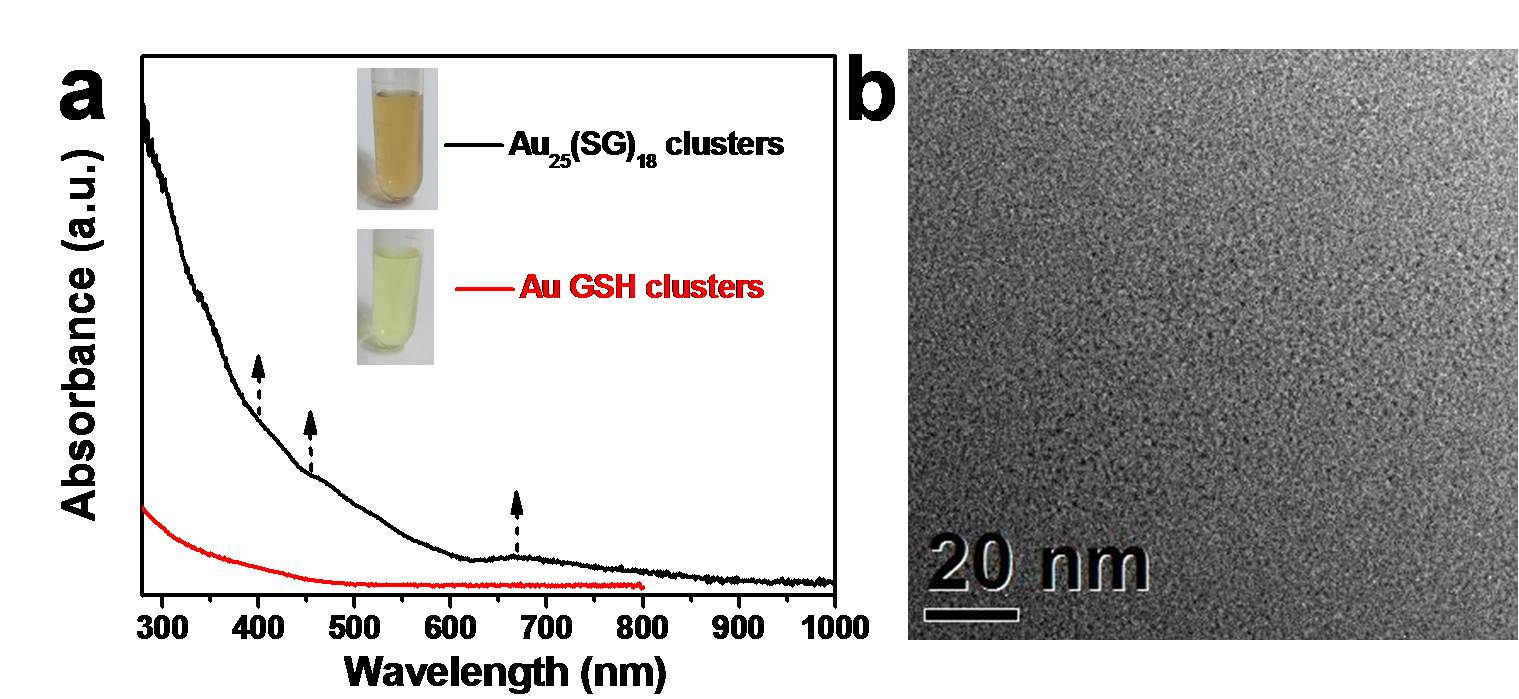


**Figure S20.** UV-vis absorption and digital photographs of Au GSH clusters and Au_25_(SG)_18_ clusters in aqueous suspension (a) and TEM image of Au_25_(SG)_18_ clusters (b).

**Note:** The obtained Au_25_(SG)_18_ clusters around 1.1 nm show similar color, size and optical absorption with characteristic peaks of other Au_25_ clusters in the most reported literatures,^42,S2-S5^ which confirm the purity of the monodispersed Au_25_(SG)_18_ clusters. Optical absorption spectrum of the Au_25_(SG)_18_ clusters shows rich features attributed to molecular transitions. The absorption at 672 nm is due to the intraband transition (HOMO-LUMO) derived from sp orbitals of Au. This position is characteristic of Au_25_ clusters and occurs in all Au_25_ molecules reported so far. ^42,S2-S5^


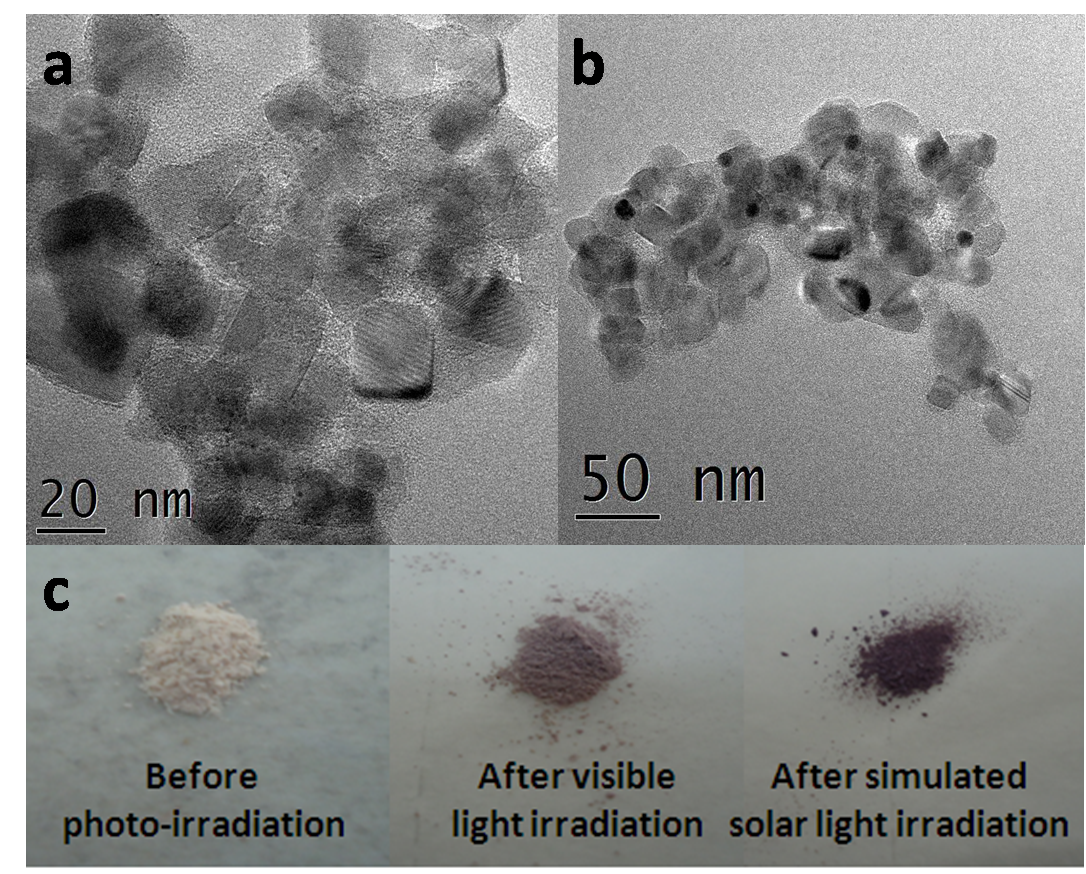


**Figure S21.** TEM images of Au_25_(SG)_18_ clusters-TiO_2_ composites (a), Au NPs-TiO_2_ composites (b) and digital photographs of Au_25_(SG)_18_ clusters-TiO_2_ composites before/after the simulated solar light and visible light photo-irradiation by using a 300 W Xenon lamp (c).

**Figure S22.** Photo-degradation of RhB over Au_25_(SG)_18_ clusters-TiO_2_ composites and Au NPs-TiO_2_ composites under the simulated solar light irradiation (a) and visible light irradiation (b) by using a 300 W Xenon lamp.

**Figure S23.** High-resolution XPS spectra of Au 4f (a), Ti 2p (b), N 1s (c) and S 2p (d); Raman (e) and FTIR (f) spectra for Au_25_(SG)_18_ clusters-TiO_2_ composites before/after the simulated solar light photo-irradiation.

**Note:** The Au 4f_5/2_ and Au 4f_7/2_ peaks with binding energies (BEs) of 87.50 eV and 83.80 eV are ascribed to metallic Au (Au^0^), and the other doublet at 88.85 eV and 85.20 eV for Au 4f_5/2_ and Au 4f_7/2_are assigned to Au^+^.^27, 43- 45^ After the photo-irradiation, Au^+^ peaks disappeared, which further evidences the photo-induced transformation of Au_25_(SG)_18_ clusters to Au NPs. Moreover, the intensity of S 2p_3/2_ peak at 163.8 eV, corresponding to the ligands features (RS−Au) in Au_25_(SG)_18_ clusters becomes weaker after the photo-irradiation, while new peaks at 169.2 and 170.8 eV, matching the BE of R−SO_3_ species are observed, suggesting that the sulfur-containing ligands undergo an oxidation process during the photo-irradiation.^45-48^ The BE shift for Ti 2p_3/2_ and decreased N 1s peaks after the photo-irradiation also provide evidences on the ligands oxidation process. Five Raman characteristic peaks at 146 (E_g_), 199 (E_g_), 399 (B_1g_), 517 (A_1g_ + B_1g_) and 639 (E_g_) cm^-1^ for Au_25_(SG)_18_ clusters-TiO_2_ and Au NPs-TiO_2_ composites can be assigned to TiO_2_.^63^ The peak at 490 cm^-1^ corresponds to S-S stretching.^49^ 1628 cm^-1^ peak of FTIR spectra corresponds to stretching vibration modes of -COOH groups from GSH ligands; 1424 cm^-1^ peak corresponds to CH_2_−S methylene scissoring (δ) in Au_25_(SG)_18_ clusters-TiO_2_ composites. Strong 1340 cm^-1^ peak is for thiosulfonate as well as weak 1079 cm^-1^ and 1042 cm^-1^ peak for R-SO_3_ in Au NPs-TiO_2_ composites.^45^


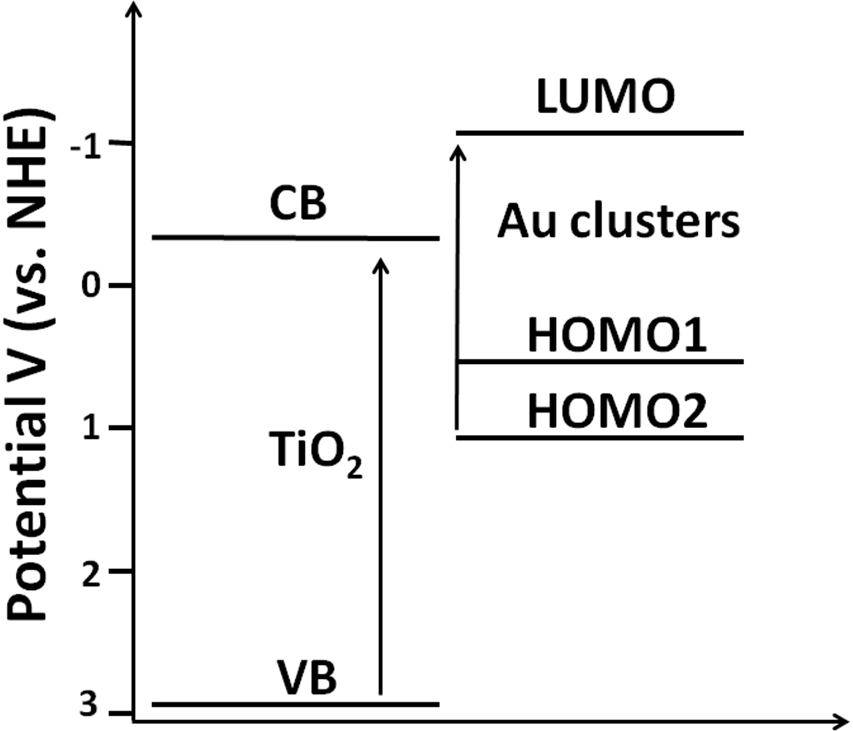


**Figure S24.** Simplified Au GSH clusters and TiO_2_ energy level diagram that was created from the data in ref 28.^28^

**Figure S25.** Emission spectra of Au GSH clusters aqueous solution adding different amount of TiO_2_. Excitation wavelength is 420 nm (3 mL of Au GSH clusters aqueous solution).


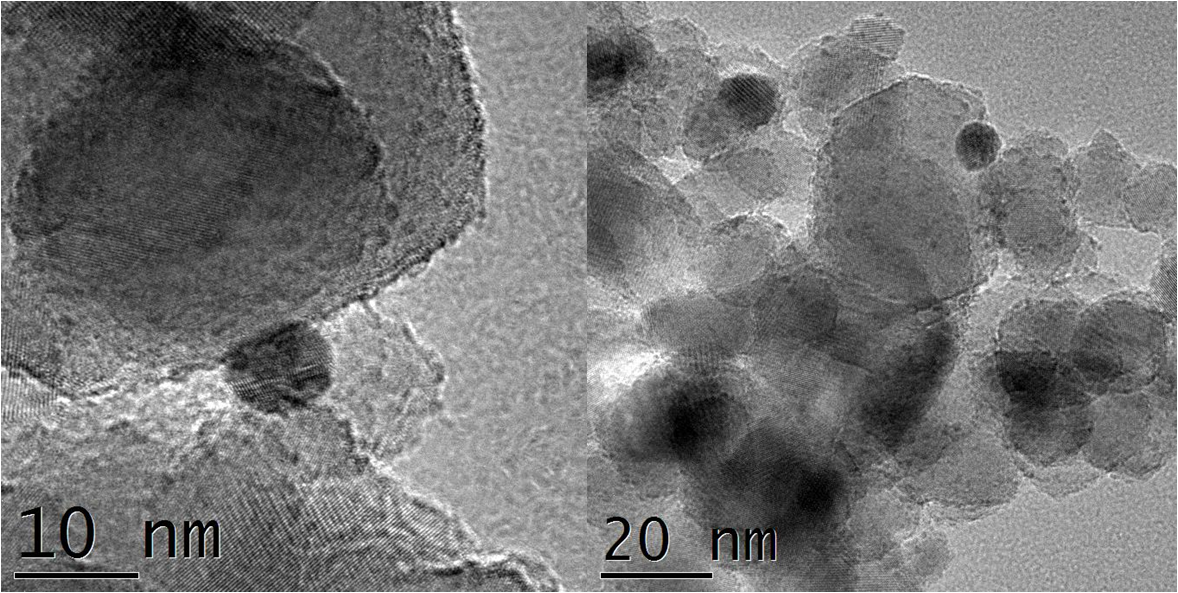


**Figure S26.**TEM images of Au GSH clusters-TiO_2_ composites after 5 h visible light photo-irradiation treatment by using a 150 W Xenon lamp, which shows the co-existence of both Au GSH clusters and Au NPs.

**Note:** Due to the fast transformation of Au GSH clusters under the simulated solar light photo-irradiation in a 300 W Xenon lamp, we utilized 150 W Xenon lamp with visible light photo-irradiation to study the Au GSH clusters transformation. After 5 h visible light photo-irradiation, the co-existence of both Au GSH clusters and Au NPs with an interfacial contact can be observed in the resulting Au GSH clusters-TiO_2_ composites. This synergistic effect between molecular-like Au GSH clusters and plasmonic Au NPs indicates a distinct photocatalytic mechanism from original Au GSH clusters-TiO_2_ composites and Au NPs-TiO_2_ composites, which involves the first transfer of electrons from clusters to the Au NPs and then to the CB of TiO_2_ in which Au NPs act as a bridging medium to reinforce the flow of electrons.^63^

**Figure S27.** Photocatalytic reduction of Cr (VI) to Cr (III) over Au GSH clusters-TiO_2_ composites, Au GSH clusters-TiO_2_ composites after 1 h visible light photo-irradiation treatment by using a 150 W Xenon lamp and Au NPs-TiO_2_ composites under the visible light irradiation by using a 300 W Xenon lamp.

**Note:** Au GSH clusters-TiO_2_ composites after 1 h visible light photo-irradiation treatment in a 150 W Xenon lamp show the co-existence of both Au GSH clusters and Au NPs, which indicates a distinct photocatalytic mechanism from original Au GSH clusters-TiO_2_ composites and Au NPs-TiO_2_ composites. The synergistic effect between molecular-like Au GSH clusters and plasmonic Au NPs leads to a specific photo-generated electrons transfer pathway which involves the first transfer of electrons from clusters to the Au NPs and then to the CB of TiO_2_.^63^ The different photocatalytic mechanism leads to the enhanced photoactivity of Au GSH clusters-TiO_2_ composites after 1 h visible light photo-irradiation treatment in a 150 W Xenon lamp as compared to original Au GSH clusters-TiO_2_ composites and Au NPs-TiO_2_ composites. It should be emphasized that although Au GSH clusters-TiO_2_ composites after 1 h visible light photo-irradiation treatment exhibit higher photocatalytic activity than both original Au GSH clusters-TiO_2_ composites and Au NPs-TiO_2_ composites, photo-stability effects of the samples during the photocatalytic process on the photocatalytic mechanism are not taken into account in this photocatalytic process. With prolonging the photo-irradiation time, the inevitable aggregation of Au GSH clusters to Au NPs leads to the varying compositions of Au GSH clusters-TiO_2_ composites, which further increases the complexity of Au GSH clusters-TiO_2_-based photocatalysis with a dynamically mutative photocatalytic mechanism during the photocatalytic reactions.


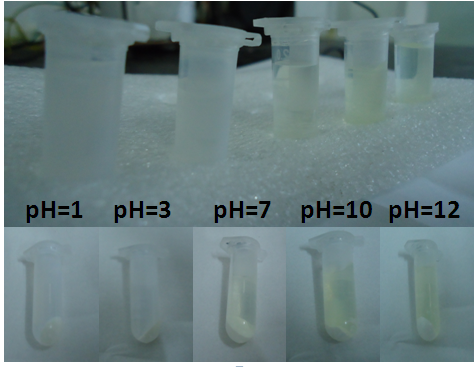


**Figure S28.** Digital photographs of the synthesis of Au GSH clusters-TiO_2_ composites under different pH value.

**Note:** We have adjusted the pH value of the Au GSH cluster solution from pH = 1 to pH = 12. 50 mg TiO_2_ was added into 2 ml Au GSH cluster solution containing 0.5 mg Au GSH clusters. The dispersion was centrifuged after stirring for 1 h. Under acid conditions, the supernatant was colorless, which means an efficient interaction between Au GSH clusters and the TiO_2_ surface. However, under neutral or basic conditions, the supernatant exhibited obviously light yellow, which suggests an insufficient interaction between Au GSH clusters and the TiO_2_ surface.

**Table S1.** Summary of the color change results of a series of controlled experiments.

|  | **Air** | **O_2_** | **N_2_** | **Vacuum** |
| --- | --- | --- | --- | --- |
| **Dark&Heat^a^** | light yellow^d^ | light yellow | light yellow | light yellow |
| **Visible light** | purple^e^ | purple | light gray ^f^ | light gray |
| **Visible light &Hole scavenger^b^** | purple | purple | light gray | light gray |
| **Visible light &Hydroxyl radical scavenger^c^** | purple | purple | light gray | light gray |
| **UV-vis light** | purple | purple | purple | purple |
| **UV-vis light &Hole scavenger** | purple | purple | light gray | light gray |
| **UV-vis light &Hydroxyl radical scavenger** | purple | purple | light gray | light gray |

^a^ The heating temperature is 100 °C.

^b^ Using ammonium formate as hole scavengers.

^c^ Using tert-butanol as hydroxyl scavengers.

^d^ The color of fresh Au GSH clusters-TiO_2_ composites.

^e^ The color of Au NPs-TiO_2_ composites, indicating Au GSH clusters aggregation in the reaction conditions.

^f^ The color of Au GSH clusters-TiO_2_ composites, indicating the inhibited aggregation of Au GSH clusters in the reaction conditions.

**References**

S1. Zhang, Y. et al. TiO_2_−graphene nanocomposites for gas-phase photocatalytic degradation of volatile aromatic pollutant: Is TiO_2_−graphene truly different from other TiO_2_−carbon composite materials? *ACS Nano* ***4*,** 7303-7314 (2010).

S2. Yao, H. On the electronic structures of Au_25_(SR)_18_ clusters studied by magnetic circular dichroism spectroscopy. *J. Phys. Chem. Lett.* ***3*,** 1701-1706 (2012).

S3. Wu, Z. et al. Thiolate ligands as a double-edged sword for CO oxidation on CeO_2_ supported Au_25_(SCH_2_CH_2_Ph)_18_ nanoclusters. *J. Am. Chem. Soc.* ***136*,** 6111-6122 (2014).

S4. Liao, L. et al. Mono-mercury doping of Au_25_ and the HOMO/LUMO energies evaluation employing differential pulse voltammetry. *J. Am. Chem. Soc.* ***137*,** 9511-9514 (2015).

S5. Shibu, E. S. et al. Ligand exchange of Au_25_SG_18_ leading to functionalized gold clusters: Spectroscopy, kinetics, and luminescence. *J. Phys. Chem. C* ***112*,** 12168-12176 (2008).
